# A software tool of digital tomosynthesis application for patient positioning in radiotherapy

**DOI:** 10.1120/jacmp.v17i2.5999

**Published:** 2016-03-08

**Authors:** Hui Yan, Jian‐Rong Dai

**Affiliations:** ^1^ Department of Radiation Oncology Cancer Hospital Chinese Academy of Medical Sciences Beijing China

**Keywords:** DTS, CBCT, GPU

## Abstract

Digital Tomosynthesis (DTS) is an image modality in reconstructing tomographic images from two‐dimensional kV projections covering a narrow scan angles. Comparing with conventional cone‐beam CT (CBCT), it requires less time and radiation dose in data acquisition. It is feasible to apply this technique in patient positioning in radiotherapy. To facilitate its clinical application, a software tool was developed and the reconstruction processes were accelerated by graphic processing unit (GPU). Two reconstruction and two registration processes are required for DTS application which is different from conventional CBCT application which requires one image reconstruction process and one image registration process. The reconstruction stage consists of productions of two types of DTS. One type of DTS is reconstructed from cone‐beam (CB) projections covering a narrow scan angle and is named onboard DTS (ODTS), which represents the real patient position in treatment room. Another type of DTS is reconstructed from digitally reconstructed radiography (DRR) and is named reference DTS (RDTS), which represents the ideal patient position in treatment room. Prior to the reconstruction of RDTS, The DRRs are reconstructed from planning CT using the same acquisition setting of CB projections. The registration stage consists of two matching processes between ODTS and RDTS. The target shift in lateral and longitudinal axes are obtained from the matching between ODTS and RDTS in coronal view, while the target shift in longitudinal and vertical axes are obtained from the matching between ODTS and RDTS in sagittal view. In this software, both DRR and DTS reconstruction algorithms were implemented on GPU environments for acceleration purpose. The comprehensive evaluation of this software tool was performed including geometric accuracy, image quality, registration accuracy, and reconstruction efficiency. The average correlation coefficient between DRR/DTS generated by GPU‐based algorithm and CPU‐based algorithm is 0.99. Based on the measurements of cube phantom on DTS, the geometric errors are within 0.5 mm in three axes. For both cube phantom and pelvic phantom, the registration errors are within 0.5 mm in three axes. Compared with reconstruction performance of CPU‐based algorithms, the performances of DRR and DTS reconstructions are improved by a factor of 15 to 20. A GPU‐based software tool was developed for DTS application for patient positioning of radiotherapy. The geometric and registration accuracy met the clinical requirement in patient setup of radiotherapy. The high performance of DRR and DTS reconstruction algorithms was achieved by the GPU‐based computation environments. It is a useful software tool for researcher and clinician in evaluating DTS application in patient positioning of radiotherapy.

PACS number(s): 87.57.nf

## I. INTRODUCTION

The modern intensity‐modulated radiation therapy (IMRT) has been widely used in radiation oncology for external treatment of cancer for many years. Due to its requirement of high conformity, the accuracy of patient positioning during treatment is critical. To achieve this goal, onboard kV image‐guided patient positioning was adopted in recent years.^1^, ^2^ This required a large amount of CB projections (370 or 670) acquired with a partial or full rotations of linac gantry in order to reconstruct a CBCT. The acquisition time of this process is about 60 s for a full rotation and 30‐40 s for a half rotation. The radiation dose for a CBCT scan is about 2‐4 cGy and the cumulative dose for a treatment consisting of 30 fractions is about 60‐120 cGy. For a 4D CBCT scan, three or four thousand CB projections are necessary, and the total dose would be more than 10 cGy per scan.^3^, ^4^, ^5^ Therefore, the use of CBCT was precautious in clinical practice.

As an alternative to this potentially harmful imaging dose, digital tomosynthesis (DTS) was introduced to provide a low‐dose imaging solution to CBCT. The advantage of DTS is that it only needs a small amount of CB projections covering a narrow scan angle, and subsequently requires less imaging dose and acquisition time. The anatomical structures on DTS are clearly viewed on focal plane in the direction of central scan angle (such as 0° or 270°), but are less clear in the other directions due to the missing projection information from the other angles. Also, the anatomical structures on DTS planes are gradually blurred as they are off the focal plane.^6^, ^7^ Since the anatomical structures on DTS can be clearly viewed on the focal plane, target shifts in 2D DTS plane can be determined accurately, but the shift in the third dimension was unclear. Therefore, two sets of DTSs are required with different central scan angles in order to determine the target shifts in the third dimension.^8^, ^9^, ^10^ Even with the limitations it is still worth investigating the applicability of DTS in daily patient positioning in radiotherapy because of the advantage of low dose and fast acquisition.

Recently DTS is introduced to 4D verification of target location due to respiration motion^11^, ^12^ and patient setup in several treatment sites including breast, lung, and liver were investigated.^13^, ^14^ As more DTS datasets are required in 4D and the other clinical treatment sites, the reconstruction efficiency of DTS become more important. In general, there are three type of DTS reconstruction algorithm: the shift‐and‐add (SAA) algorithm,^(15)^ the simultaneous algebraic reconstruction technique (SART),^(16)^ and a filtered back‐projection algorithm based on the Feldkamp‐Davis‐Kress (FDK) algorithm.^(17)^ The SAA algorithm is originally developed for acquisition system with source and detector moving in opposite parallel directions, and is not suitable for current C‐arm on‐board imaging devices. The SART needs multiple iterations in order to converge to an optimal result and is not clinically practical. The FDK algorithm is designed for cone‐beam tomography and is used as standard approach for CBCT reconstruction in most commercial systems. Recently, GPU‐based FDK algorithms were introduced for acceleration purpose.^18^, ^19^ In principle, the FDK algorithm for CBCT reconstruction could be used for reconstructing DTS. However, unlike CBCT, DTS planes are reconstructed in a specific orientation (such as coronal or sagittal) instead of axial plane. Besides, there was another type of DTS‐ODTS to be reconstructed from DRRs for registration purpose.^20^, ^21^ Therefore, the FDK algorithms originally developed for CT/CBCT reconstruction should be adjusted for the image orientation of DTS application. To facilitate the implementation of DTS application, a software tool was specially developed for research and clinical purposes.

Currently there are no commercial DTS systems developed for clinical purpose. But some are available for research society. Varian (Varian Medical Systems, Palo Alto, CA) provided a DTS software tool that allows for the creation and registration of DTS‐based datasets. It consists of three separate modules: 1) reconstruction of ODTS, 2) reconstruction of RDTS, and 3) registration of ODTS and RDTS to provide 3D shifts. Several institutes tested their data on this software tool in order to evaluate the capability of DTS in clinical setting.^21^, ^22^ Their result are convincing, but still need more investigation. Another software developed for this purpose is DART (Digital Tomosynthesis Applications in Radiotherapy Toolkit; Department of Radiation Oncology, Huntsman Cancer Hospital, Salt Lake City, UT).^(23)^ The software allows for DTS reconstruction using three different reconstruction methods. The system is mainly programmed on the MATLAB (version 7.5, MathWorks Inc., Natick, MA), and only accepts Theraview (Acceletronics, Exton, PA) EPID images and Pinnacle (Philips Healthcare, Andover, MA) DRRs as input. The software is mainly built for MVCT in evaluating its performance against KVCT.

The purpose of this project is to develop a software tool in supporting DTS application in clinical setting. This software tool is developed on an open‐source environment and easily deployed in various hardware platforms. High performance GPU‐based reconstruction algorithms are implemented and could run on any type of commercial computation platform. The standard clinical data can be easily imported and exported with the implementation of various image filters. In the Materials & Methods section, the flowchart of DTS application is briefly introduced, followed by the description of DRR and DTS reconstruction algorithm and their GPU implementation. The software tool is evaluated by cube phantom and pelvic phantom for geometric and registration accuracy. The image quality and reconstruction efficiency are investigated on the experiments of pelvic phantom. The accuracy and performance of this software tool are summarized in the Results section. Finally, the benefit and disadvantage of this software tool are discussed in the Discussion section.

## II. MATERIALS AND METHODS

### A. Software design

**Figure 1:**
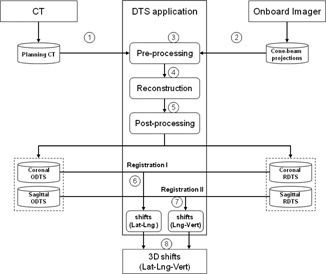
The scheme of DTS reconstruction and registration process. Each step is labeled by a number which represents the order of this module in the whole process.

The main functions of this application are programmed using visual C++ (version 9, Microsoft Corp, Redmond, WA). The graphical user interface (GUI) is developed with Java GUI widget toolkit, Swing (Oracle Corporation, Redwood Shores, CA). Each function can either run independently in command line or called by user interface. The general workflow of this DTS application is illustrated in Fig. 1 which each step is labeled by a number. At first, planning CT is imported from CT scanner in Step 1 and CB projections are transferred from onboard imager (OBI) in Step 2. Both sets of raw data are processed in preprocessing module in Step 3 in which the parameters required by reconstruction algorithms are collected. Then in Step 4, DRR is calculated from planning CT followed by a reconstruction process of RDTS. And ODTS is reconstructed directly from CB projections. The DTS planes in coronal view are reconstructed from CB projections centered at 0°, while the DTS planes in sagittal view are reconstructed from CB projections centered at 270°. The reconstructed DTS planes are saved as DICOM files in postprocessing module in Step 5. Later, these DICOM files are imported to registration software (Varian offline review, Varian Medical Systems). The target shifts in lateral and longitudinal axes are determined by matching coronal ODTS and coronal RDTS in coronal view in Step 6, while the target shifts in longitudinal and vertical axes are determined by matching sagittal ODTS and sagittal RDTS in sagittal view in Step 7. Finally, the target shifts in three dimensions is obtained in Step 8.

**Figure 2:**
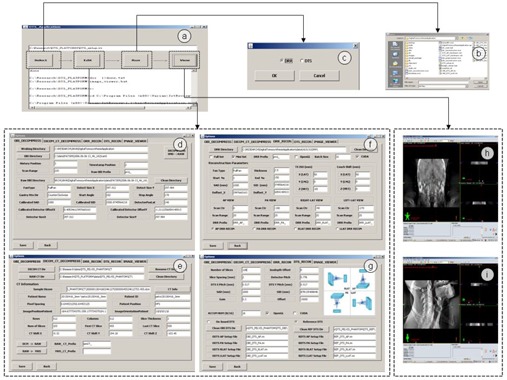
The user interface of DTS software tool: (a) main window; (b) window of file selection; (c) window of DRR and DTS reconstructions selection; (d) window of parameter configuration for cone‐beam projection decompression; (e) window of parameter configuration for planning CT decompression; (f) window of parameter configuration for DRR reconstruction; (g) window of parameter configuration for DTS reconstruction; (h) demonstration of image registration between coronal ODTS and coronal RDTS; (h) demonstration of image registration between sagittal ODTS and sagittal RDTS.

The software user interface is shown in Fig. 2. The main window is shown in Fig. 2(a) from which user can select setup file, edit parameters, reconstruct image, and register images. Initially, a setup file is selected, as shown in Fig. 2(b). This setup file contains general information required by DTS application, and their default values are set at the beginning. Since there are many parameters related to DTS application and different categories of parameters are predefined. These categories of parameters are those related to decompression of CB projections (Fig. 2(d)), decompression of DICOM CT (Fig. 2(e)), DRR reconstruction (Fig. 2(f)), and DTS reconstruction (Fig. 2(g)). When these parameters are properly selected, images can be generated correctly. There are two types of images, DRR and DTS, to be generated, as shown in Fig. 2(c). DRR images are reconstructed prior to the reconstruction of RDTS, while ODTS is reconstructed directly from on‐board cone‐beam projections. When both ODTS and RDTS datasets are prepared, they are registered to each other in Varian offline review software. As an example, the image registration between coronal ODTS and coronal RDTS is shown in Fig. 2(h), while the image registration between sagittal ODTS and sagittal RDTS is shown in Fig. 2(i).

### B. Reconstruction

#### B.1 DRR reconstruction

General ray‐casting algorithm is used to calculate DRR from planning CT in this software. The principle of ray‐casting algorithm is to find intersection of a ray with voxel of a CT volume. Rays are traced through the CT volume with the origin of the ray placed at the X‐ray source and the direction of the ray determined by the pixel of the detector.^24^, ^25^ Because hundreds of thousands of rays are calculated for creating a single DRR, the computation cost on general‐purpose CPU is higher. To improve its performance, two dedicated GPU‐based algorithms are implemented in this software. One is developed on OpenGL (Open Graphics Library) environment which is industry‐standard application programming interface (API) used to achieve hardware‐accelerated rendering. Another is developed on CUDA (the Computer Unified Device Architecture) environment, which is a parallel computing and programming platform called NVIDIA (Nvidia Corporation, Santa Clara, CA). The reason for providing both algorithms is that OpenGL is a cross‐platform API and could potentially run on any general‐purpose computers, but may be not well designed for parallel computation. CUDA is specially designed for parallel computation, but can only work on hardware supported by Nvidia Company. The user can make the best use of the two algorithms based on their hardware setting. The detail descriptions of OpenGL‐based DRR algorithms can be referenced in our previous publications.^26^, ^27^ The CUDA‐based DRR algorithms are revised from the DRR codes provided by the open‐source software, Plastimatch (http://www.plastimatch.org/publications.html), which is developed by multiple groups (Department of Radiation Oncology, Massachusetts General Hospital, Boston and Electrical and Computer Engineering Department, Drexel University, Philadelphia, PA). The description of CUDA‐based DRR algorithms provided by Plastimatch can be found in their publications.^(28)^

The pseudocodes for DRR reconstruction implemented on three computation environments are illustrated in Appendix A. They basically share the same workflow but differ in the data storage and ray‐tracing process. The first two steps are the same for the three algorithms. For ray‐tracing process, the CPU‐based algorithm first applies a rotational matrix on CT volume in simulating rotation of linac gantry around patient. Then CPU‐based algorithm calculates intersecting points of ray with 2D slice of CT volume, interpolates the value of intersecting points along the ray, sums the values of intersecting points on the ray, and assigns the summed value to the intersecting pixel on kV detector. For the OpenGL‐based algorithm, it first allocates 3D array for CT volume and 2D arrays for DRRs in texture memory, and then copies CT volume to 3D texture memory in GPU memory. For each DRR, OpenGL‐based algorithm applies rotational matrix on CT volume in simulating rotation of linac gantry around patient. For all coronal planes of rotated CT volume, applies projecting matrix on them and sums the projected pixels of CT volume in the frame buffer of GPU. If the content of frame buffer is full, copy it to 2D array in CPU memory. Unlike the OpenGL‐based algorithm, the CUDA‐based algorithm first determines the intersecting pixel of ray on detector plane. Then it determines intersecting points of ray with CT volume and gets their CT values from texture memory. Finally, these CT values are summed and assigned to the intersecting pixel stored in 2D array in GPU memory. After all pixels of a DRR are processed, the 2D array stored in GPU memory is copied to CPU memory and exported as DICOM files.

#### B.2 DTS reconstruction

**Figure 3:**
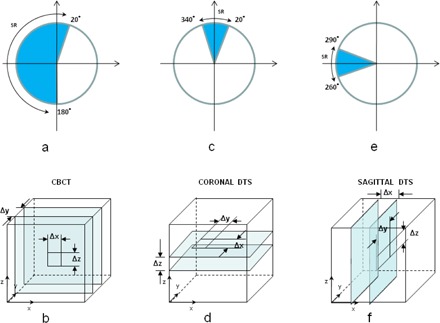
The comparison of CBCT and DTS in scan range of CB projections and the orientation of reconstructed planes: (a) scan range (200°) of CB projections for CBCT; (b) definition of CBCT plane in transversal view, Δx and Δz represent the pixel sizes, while Δy represents the slice thickness; (c) scan range (40°) of CB projections for coronal DTS; (d) definition of coronal DTS plane in coronal view, Δx and Δy represent the pixel sizes, while Δz represents the slice thickness; (e) scan range (40°) of CB projections for sagittal DTS; (d) definition of sagittal DTS plane in sagittal view, Δy and Δz represent the pixel sizes, while Δx represents the slice thickness.

General FDK algorithm is used to calculate DTS from CB projections or DRRs in this software. The principle of FDK algorithm has been available for many years.^16^, ^17^ In principle, the voxel of DTS in 3D first identifies the intersecting pixel on 2D detector by tracing ray passing through it. Then the value of intersecting pixel contributes its value to the voxel of DTS. The difference between DTS and CBCT in the scan range of CB projections and the orientation of reconstructed planes are comparatively demonstrated in Fig. 3. For CBCT using Varian OBI, the total scan range is 200° for full‐fan CBCT scan as shown in Fig. 3(a) and the reconstructed planes are in transversal view. The pixel size in CBCT plane is Δx and Δz, and slice thickness is Δy as shown in Fig. 3(b). For coronal DTS, the total scan range is 40° and the central angle is 0° as shown in Fig. 3(c), and the reconstructed planes are in coronal view. The pixel size in coronal DTS plane is Δx and Δy, and slice thickness is Δz, as shown in Fig. 3(d). For sagittal DTS, the total scan range is 40° and the central angle is 270° as shown in Fig. 3(e) and the reconstructed planes are in sagittal planes. The pixel size in sagittal DTS plane is Δy and Δz, and slice thickness is Δx as shown in Fig. 3(f). Note that the resolutions of CBCT and DTS planes are different in three dimensions.

The reconstruction of DTS reconstruction on general‐purpose CPU is time‐consuming. To improve this performance, two dedicated GPU‐based algorithms are implemented. One is based on OpenGL and another is based on CUDA. Both algorithms provide functions to reconstruct coronal DTS planes from CB projections/DRRs centered at 0° and sagittal DTS planes from CB projections/DRRs centered at 270°. For half‐fan mode, two sets of coronal DTS planes are first reconstructed from CB projections/DRRs centered at 0° and 180°, and stitched together to form a final DTS set. The same process is performed for sagittal DTS planes. This is because the CB projections acquired in half‐fan mode only provides half side of imaging object, and the other half side information of object is provided by the CB projections acquired in the opposite direction. Therefore, the DTSs in both sides should be combined together. The detail descriptions of OpenGL‐based DTS algorithms can be referenced in our previous publications.^26^, ^27^ The CUDA‐based DTS algorithms are revised from the FDK codes provided in the open source software, Plastimatch. The most detail of CUDA‐based FDK algorithms can be found in their publications.^(28)^

The pseudocodes for DTS reconstruction implemented on three computation environments are comparatively illustrated in Appendix B. They basically share the same workflow, but differed in the operations of data storage and back‐projecting process. The first three steps are the same for the three algorithms: Step 1 allocating 3D array for DTS volume and 2D array for CB projections/DRRs in CPU memory, Step 2 loading CB projection/DRR into 2D array in CPU memory, and Step 3 performing logarithm conversion and FFT filtering on 2D CB projection/ DRR. For back‐projecting process, the CPU‐based algorithm calculates intersecting point of ray in 2D detector, interpolates the value of intersecting point on 2D CB projection/DRR, and adds it to the value of voxel of DTS along the ray. For the OpenGL‐based algorithm, it first allocates 3D array for DTS volume and 2D array for CB projections/DRRs in GPU texture memory, and copies 2D CB projection/DRR into a set of 2D textures in GPU memory. Then for each CB projection/DRR, the algorithm applies translational and rotational matrixes on 2D texture and projects it on the frame buffer of GPU. If the content of frame buffer is full, transfer the content of frame buffer to 2D array in CPU memory. Similarly, the CUDA‐based algorithm first allocates 3D array for DTS volume and 2D array for CB projections/DRRs in GPU memory, and then transfers 2D CB projections/DRRs into 2D texture memory in GPU. Then for each voxel of DTS, it finds the index of intersecting pixel at 2D CB projections/DRRs, gets the value of pixel from 2D array in texture memory, and adds it to the value of voxel of DTS along the ray. As the final step, all three algorithms exports 3D array storing DTS volume into a series of DICOM files containing 2D DTS planes.

### C. Image registration

Varian offline review software is used for registering ODTS with RDTS. The Varian offline review software provides both manual and automated matching approaches for image registration and tools for image analysis. As DTS reconstruction accomplished, DTS planes are saved as DICOM files and stored at a given locations. Meanwhile, the path of DICOM files and the patient information are automatically updated in the setup file of Varian offline review software by postprocessing module. When users launch Varian offline review software and choose the setup file, both sets of ODTS and RDTS are load from their default locations. As illustrated in Fig. 1, four sets of DTSs are prepared for each patient. They are coronal ODTS, coronal RDTS, sagittal ODTS, and sagittal RDTS. Two registration sessions are performed in order to determine target shift in three dimensions. In the first registration session, the target shifts in lateral and longitudinal axes are determined by matching coronal ODTS and coronal RDTS in three views. Note that since the coronal DTS are reconstructed from CB projections/DRRs centered at 0°, the conspicuity of anatomical structures is better in coronal view, poor in sagittal view, and worse in transversal view. Therefore, the registration results in lateral and longitudinal axes are used in first registration session. In the second registration session, the target shifts in longitudinal and vertical axes are determined by matching sagittal ODTS and sagittal RDTS in three orthogonal views. Note that, since the sagittal DTS are reconstructed from CB projections/DRRs centered at 270°, the conspicuity of anatomical structures is better in sagittal view, poor in coronal view, and worse in transversal view. Therefore, the registration results in longitudinal and vertical axes are used in second registration session. As the target shifts in longitudinal axis are measured twice in two registration sessions, their average is used as the final value. Note that, in this study, only manual registration was employed.

### D. Validation

To validate the performance of this software tool, comprehensive tests were performed. These tests include the evaluation of geometric accuracy, correlation analysis, registration error, and reconstruction efficiency. A water‐equivalent cube phantom and a pelvis phantom are used for all tests. Both phantoms are scanned on a Siemens SOMATOM Definition AS CT Scanner (Siemens Medical Solutions USA Inc., Malvern, PA). CT volume is reconstructed with slice thicknesses of 1.0 mm and pixel sizes of 1.0 mm×1.0 mm in plane. The abdomen CT scan protocol (with

80 KV and exposure 560 mA) is employed. The phantom is originally positioned on the couch of CT room. The metal markers are attached to phantom surface and aligned with the CT room laser on the wall. After CT scanning, phantom is transferred to the treatment room. The same CT scanning position is reproduced based on the metal markers and room laser. An on‐board kV device (OBI, Varian medical system, Palo Alto, CA) on a Varian Novalis TX linear accelerator is used to acquire a full set of cone‐beam (CB) projections. A CBCT protocol (standard‐dose head, full‐fan with bowtie filter) is used for cube phantom and another CBCT protocol (pelvis spot light, full‐fan with bowtie filter) is used for pelvic phantom. In the full‐fan scan mode the gantry rotated from 20° to $-180^{\circ }$ counterclockwise with the speed of 6° per second and covered 200° scan angles. This resulted in 375 CB projections and the average angle spacing is 0.53°. The image size of CB projection is 397 mm×298 mm (image resolution of 512×384) and pixel size is 0.776 mm. The DTS was reconstructed from a subset of CB projections and in the dimensions of 512×512×256 with the nominal voxel size (0.5 mm) in three dimensions.

#### D.1 Geometric accuracy

Geometric accuracy was evaluated by measuring length of cube phantom on ODTS. Here, coronal and sagittal ODTSs of cube phantom are reconstructed from CB projections using three types of algorithms. For each algorithm, four set of ODTSs are reconstructed from four sets of CB projections covering scan range of 20°, 40°, 60°, and 80°. The lengths of cube phantom in lateral and longitudinal axes are measured on coronal ODTS, and the lengths of cube phantom in longitudinal and vertical axes are measured on sagittal ODTS. The measured lengths of cube phantom on ODTS are compared with the actual lengths (5 cm) for errors. For each length of cube phantom, five measurements are performed and their differences from the actual length of cube phantom are calculated. The average differences and their standard deviations are reported.

#### D.2 Correlation analysis

The similarity between DRRs reconstructed by CPU‐based and the GPU‐based algorithms were analyzed. The DRRs reconstructed by CPU‐based algorithm is assumed to be the standard images, while the DRR generated by GPU‐based algorithms may come with certain degradation. The correlation coefficient between DRRs generated by GPU‐based and CPU‐based algorithms measures their similarity. The pelvis phantom is used for the test. Three sets of DRRs are generated by the three types of algorithms using the same acquisition setting of CB projections. This resulted in about 375 DRRs covering scan angle of 200°. The correlation coefficient between DRRs generated by GPU‐based and CPU‐based algorithms are calculated and analyzed. The subtraction images between DRRs generated by CPU‐based and GPU‐based algorithms are calculated, and their means and standard deviations are analyzed. Similarly, three sets of DTSs consisting of 128 slices are generated by CPU‐based algorithm and two GPU‐based algorithms. The correlation coefficient between DTSs generated by CPU‐based and GPU‐based algorithms are calculated and analyzed. The subtraction images between DTSs generated by CPU‐based and GPU‐based algorithms are calculated, and their means and standard deviations are analyzed.

#### D.3 Registration accuracy

Registration accuracy was evaluated by cube and pelvis phantoms in simulating patient shifting in a given amount. The target shifts are determined by matching ODTS with RDTS and compared with known amount for registration errors. CUDA‐based DRR and DTS reconstruction algorithms are used in this test. The displacements of patient are mimicked by shifting planning CT in reconstruction software a certain amount (1 mm, 3 mm, and 5 mm) from its origin in lateral, longitudinal, and vertical axes, respectively. Then DRRs are generated and followed by RDTS reconstruction. The displacements of pelvis phantom in lateral and longitudinal axes are determined by matching coronal ODTS with coronal RDTS, and the displacements of pelvis phantom in longitudinal and vertical axes are determined by matching sagittal ODTS with sagittal RDTS. The measured displacements of pelvis phantom on DTS are compared with the known amounts (1 mm, 3 mm, and 5 mm) for registration errors. The errors in lateral and longitudinal axes are determined from coronal registration process, while the errors in longitudinal and vertical axes are determined from sagittal registration process. For each test of displacement, three sets of DTS are generated from three sets of CB projections/DRRs covering three scan ranges of 20°, 40°, and 60°. For each displacement in an axis, five measurements are performed in both coronal and sagittal registration processes. The average registration errors and standard deviations are calculated and analyzed. Note that all DTSs in this study are reconstructed in the fine resolution with nominal voxel sizes of 0.5 mm×0.5 mm×0.5 mm in three dimensions.

#### D.4 Reconstruction efficiency

The reconstruction efficiency was evaluated by comparing the total processing times of CPU‐based algorithms and GPU‐based algorithms for a given reconstruction task of a head and neck case. The CT data of head and neck case are in the dimensions of 512×512×150 with pixel size 1.0 mm×1.0 mm×2.5 mm. The sizes of DRR are in the dimensions of 512×384 with pixel size 0.776 mm×0.776 mm. The dimension of DTS slice is 512×512 with pixel size 0.5 mm in plane and slice thickness 1.0 mm. Three sets of DRRs are generated from CT data by three types of algorithms and the total number of DRRs is 658. Four sets of DTSs consisting of the slice number 32, 64, 128, and 256 are reconstructed for a given scan range. Three scan ranges 30°, 45°, and 60° consisting of approximately 55, 85 and 115 CB projections are tested. Note that the processing time on each DTS reconstruction task consists of reconstruction time (purely on computation of DRR or DTS) and overhead time (data preprocessing, transferring, and postprocessing). To better quantify the performance of GPU algorithms, the reconstruction time and overhead time are reported and analyzed separately. All tests are performed on a DELL Precision T5810 computer, equipped with Intel(R) Xeon(R) E5 CPU at 3.50 GHz and 16.0GB RAM, and NVIDIA Quadro K4200 with 1344 CUDA cores and 4GB 256‐bit GDDR5.

## III. RESULTS

### A. Geometric accuracy

The lengths of cube phantom on DTS are measured in three axes and their deviations from actual length 50 mm are calculated and compared in Table 1. For three types of algorithms the lengths of cube phantom measured from ODTSs are compared. For CPU‐based algorithm, the average geometric errors measured from four sets of ODTSs in different scan ranges are 0.22±0.15 mm, 0.21±0.08 mm, and 0.22±0.09 mm in lateral, longitudinal, and vertical axes, respectively. For OpenGL‐based algorithm, the average geometric errors are 0.12±0.05 mm, 0.45±0.06 mm, and 0.17±0.09 mm in three axes, while for CUDA‐based algorithm the average geometric errors are 0.11±0.05 mm, 0.31±0.08 mm. and 0.11±0.01 mm in three axes. For different scan ranges, the lengths of cube phantom measured from ODTS are also compared. For scan range of 20°, the average geometric errors measured from three sets of DTSs generated by three types of algorithms are 0.21±0.11 mm, 0.43±0.12 mm, and 0.16±0.11 mm in three axes. For scan range of 40° the geometric errors are 0.23±0.17 mm, 0.26±0.11 mm, and 0.13±0.05 mm. For scan range of 60°, the geometric errors are 0.13±0.05 mm, 0.33±0.15 mm, and 0.13±0.05 mm, and for scan range of 80° the geometric errors are 0.11±0.02 mm, 0.26±0.15 mm, and 0.16±0.11 mm. The geometric errors measured in three axes are compared over all measurements regardless of scan range and algorithm type. The overall average geometric errors are 0.15±0.09 mm, 0.31±0.12 mm, and 0.16±0.08 mm in lateral, longitudinal, and vertical axes, respectively. The maximum errors are 0.43 mm, 0.51 mm, and 0.33 mm in lateral, longitudinal, and vertical axes, respectively.

**Table 1:** Geometric errors of DTS for cube phantom

	*Measured Lengths (mm)*					
*Scan Ranges*	*CPU‐based Algorithm*	*OpenGL‐based Algorithm*	*CUDA‐based Algorithm*
	*(LAT, LNG)*	*(LNG, VRT)*	*(LAT, LNG)*	*(LNG, VRT)*	*(LAT, LNG)*	*(LNG, VRT)*
20°	(50.3, 50.2)	(50.4, 50.3)	(50.1, 49.5)	(49.5, 50.1)	(49.8, 49.7)	(49.5, 49.9)
40°	(49.6, 49.7)	(49.9, 50.1)	(50.1, 49.5)	(49.7, 49.8)	(50.1, 49.9)	(49.7, 50.1)
60°	(50.1, 50.2)	(50.1, 49.8)	(50.2, 49.6)	(49.4, 49.9)	(50.1, 49.7)	(49.8, 50.2)
80°	(50.1, 50.1)	(50.0, 50.3)	(50.1, 49.6)	(49.7, 49.7)	(50.1, 49.5)	(49.9, 50.1)

LAT=lateral;LNG=longitudinal;VRT=vertical.

### B. Correlation analysis

**Figure 4:**
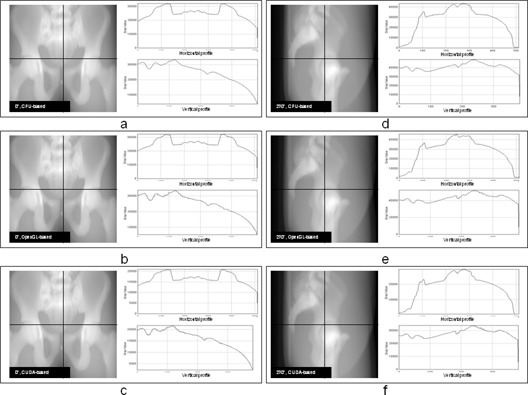
The illustration of DRR reconstructed by three types of algorithms: (a) DRRs at 0° reconstructed by CPU‐based algorithm; (b) DRRs at 0° reconstructed by OpenGL‐based algorithm; (c) DRRs at 0° reconstructed by CUDA‐based algorithm; (d) DRRs at 270° reconstructed by CPU‐based algorithm; (e) DRRs at 270° reconstructed by OpenGL‐based algorithm; (f) DRRs at 270° reconstructed by CUDA‐based algorithm. The horizontal and vertical profiles are shown in the subplots at the right side of each DRR. The locations of horizontal and vertical profiles are represented by the solid lines on each DRR.

The DRRs generated by three types of algorithms are comparatively demonstrated in Fig. 4. The three plots on the left column of Fig. 4 are DRRs at 0° generated by CPU‐based algorithm (Fig. 4(a)), OpenGL‐based algorithm (Fig. 4(b)), and CUDA‐based algorithm (Fig. 4(c)). The three plots on the right column of Fig. 4 are DRRs at 270° generated by CPU‐based algorithm (Fig. 4(d)), OpenGL‐based algorithm (Fig. 4(e)), and CUDA‐based algorithm (Fig. 4(f)). The horizontal and vertical profiles are shown in the two subplots at the right side of each DRR. The locations of these profiles are indicated by the lines displayed on DRRs. There is no visible difference between DRRs generated by GPU‐based and CPU‐based algorithms according to the comparison between their profiles. The only noticeable difference is that the profiles of DRRs generated by GPU‐based algorithm are less smooth than those of DRRs generated by CPU‐based algorithm. The average correlation coefficient is 0.99 between GPU‐based DRRs and CPU‐based DRRs. The DRR images are then rescaled to (0, 255) and the subtraction images between GPU‐based DRRs and CPU‐based DRRs are generated. The absolute means and standard deviations of subtraction images are 0.96±0.72 for OpenGL‐based algorithm and 0.93±0.64 for CUDA‐based algorithm. The relative means and standard deviations of subtraction images for DRRs are 0.3%±0.2% for both GPU‐based algorithms.

**Figure 5:**
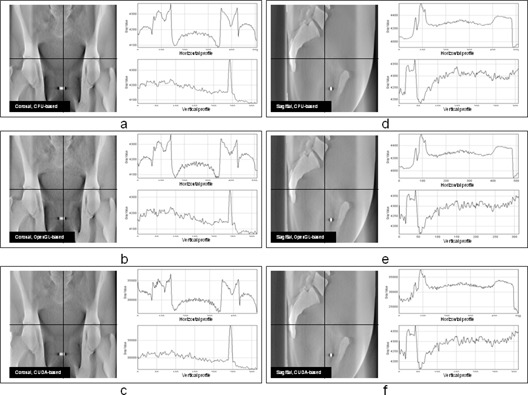
The illustration of DTS reconstructed by three types of algorithms: (a) DTSs at 0° reconstructed by CPU‐based algorithm; (b) DTSs at 0° reconstructed by OpenGL‐based algorithm; (c) DTSs at 0° reconstructed by CUDA‐based algorithm; (d) DTSs at 270° reconstructed by CPU‐based algorithm; (e) DTSs at 270° reconstructed by OpenGL‐based algorithm; (f) DTSs at 270° reconstructed by CUDA‐based algorithm. The horizontal and vertical profiles are shown in the subplots at the right side of each DTS. The locations of horizontal and vertical profiles are represented by the solid lines on each DTS.

The DTSs generated by three types of algorithms are comparatively demonstrated in Fig. 5. The three plots on the left column of Fig. 5 are coronal DTSs generated by CPU‐based algorithm (Fig. 5(a)), OpenGL‐based algorithm (Fig. 5(b)), and CUDA‐based algorithm (Fig. 5(c)). The three plots on the right column of Fig. 5 are sagittal DTSs generated by CPU‐based algorithm (Fig. 5(d)), OpenGL‐based algorithm (Fig. 5(e)), and CUDA‐based algorithm (Fig. 5(f)). The horizontal and vertical profiles are shown in the two subplots at the right side of each DTS. The locations of horizontal and vertical profiles are indicated by the lines displayed on DTSs. There is a minor difference between DRRs generated by GPU‐based and CPU‐based algorithms according to the comparison between their profiles. The profiles of DTSs generated by GPU‐based algorithm shows magnified noise compared with those of DTSs generated by CPU‐based algorithms. The average correlation coefficient is 0.99 between DTSs generated by GPU‐based and CPU‐based algorithms. The DTS images are then rescaled to (0, 255) and the subtraction images between DTSs generated by GPU‐based and CPU‐based algorithms are generated. The absolute means and standard deviations of subtraction images are 1.16±1.12 for OpenGL‐based algorithm and 1.13±1.04 for CUDA‐based algorithm. The relative means and standard deviations of subtraction images for DTSs are 0.4%±0.4% for both GPU‐based algorithms.

### C. Registration accuracy

**Figure 6:**
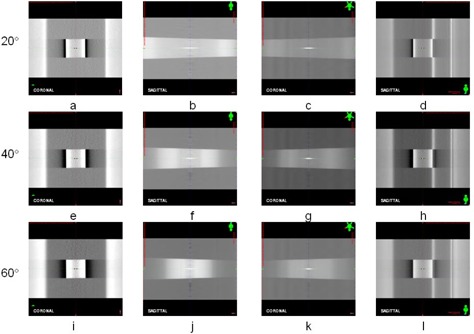
The ODTSs of cube phantom reconstructed from CB projections in three scan ranges: the coronal ODTS shown in coronal view (a) and in sagittal view (b), reconstructed in scan range 20°; (c) the sagittal ODTS shown in coronal view (c) and in sagittal view (d), reconstructed in scan range 20°; (e) the coronal ODTS shown in coronal view (e) and in sagittal view (f), reconstructed in scan range 40°; the sagittal ODTS shown in coronal view (g) and in sagittal view (h), reconstructed in scan range 40°; the coronal ODTS shown in coronal view (i) and in sagittal view (j), reconstructed in scan range 60°; the sagittal ODTS shown in coronal view (k) and in sagittal view (l), reconstructed in scan range 60°.

For cube phantom, the ODTSs reconstructed from CB projections in three scan ranges are comparatively shown in Fig. 6 in coronal and sagittal views for scan ranges 20°, 40°, and 60°. The ODTS in transversal view is not shown here since the anatomical structures are blurred and not useful for registration purpose. The registration errors of cube phantom on DTS for three displacements (1 mm, 3 mm, and 5 mm) are measured and compared in Table 2. Without introduction of displacement of cube phantom, the registration errors are summarized in the first row of the table. These errors indicated the random errors introduced by human operators, and their means and standard deviations are 0.06±0.05 mm, 0.08±0.02 mm, and 0.10±0.02 mm in lateral, longitudinal, and vertical axes, respectively. For different scan ranges, the registration errors are compared for all displacements of cube phantom. For scan ranges 20°, the means and standard deviations are 0.15±0.18 mm, 0.15±0.13 mm, and 0.15±0.07 mm in lateral, longitudinal, and vertical axes, respectively. For scan range 40° the registration errors are 0.08±0.06 mm, 0.11±0.08 mm, and 0.22±0.14 mm in three axes, and for scan range of 60° the registration errors are 0.13±0.11 mm, 0.11±0.07 mm, and 0.18±0.09 mm in three axes. For different displacements of cube phantom, the registration errors are compared for all scan ranges. For 1 mm displacement of phantom, the mean registration errors and their standard deviations are 0.10±0.08 mm, 0.17±0.12 mm, and 0.21±0.13 mm in lateral, longitudinal, and vertical axes, respectively. For 3 mm displacement, the registration errors are 0.13±0.16 mm, 0.08±0.08 mm, and 0.17±0.08 mm in three axes, and for 5 mm displacement their values are 0.12±0.08 mm, 0.12±0.05 mm, and 0.18±0.13 mm in three axes.

**Figure 7:**
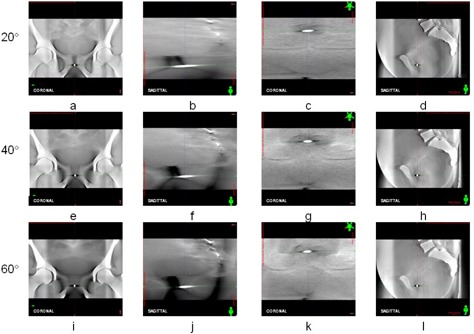
The ODTSs of pelvis phantom reconstructed from CB projections in three scan ranges: the coronal ODTS shown in coronal view (a) and in sagittal view (b), reconstructed in scan range 20°; the sagittal ODTS shown in coronal view (c) and in sagittal view (d), reconstructed in scan range 20°; the coronal ODTS shown in coronal view (e) and in sagittal view (f), reconstructed in scan range 40°; the sagittal ODTS shown in coronal view (g) and in sagittal view (h), reconstructed in scan range 40°; the coronal ODTS shown in coronal view (i) and in sagittal view (j), reconstructed in scan range 60°; the sagittal ODTS shown in coronal view (k) and in sagittal view (l), reconstructed in scan range 60°.

For the pelvis phantom, the ODTSs reconstructed from CB projections in three scan ranges are comparatively shown in Fig. 7 in the coronal and sagittal views for scan ranges 20°, 40°, and 60°. The ODTS in transversal view is not displayed here since the anatomical structures are blurred and not useful for registration purpose. The registration errors of pelvis phantom on DTS for three displacements (1 mm, 3 mm, and 5 mm) are measured and compared in Table 3. Without displacement of phantom, the registration errors are reported in the first row of Table 3. The mean registration error for zero displacement is 0.13±0.05 mm, 0.12±0.05 mm, and 0.13±0.05 mm in lateral, longitudinal, and vertical axes, respectively. The registration errors are compared for different scan ranges. For scan ranges 20°, the mean registration errors are 0.14±0.10 mm, 0.19±0.05 mm, and 0.22±0.08 mm in lateral, longitudinal, and vertical axes, respectively. For scan range 40°, the mean registration errors are 0.15±0.11 mm, 0.18±0.05 mm, and 0.20±0.10 mm in three axes, and for scan range 60° their values are 0.11±0.06 mm, 0.21±0.08 mm, and 0.16±0.08 mm in three axes. For different displacement of phantom, registration errors are compared for all scan ranges. For displacement of 1 mm, the mean registration errors are 0.10±0.07 mm, 0.16±0.08 mm, and 0.13±0.05 mm in lateral, longitudinal, and vertical axes, respectively. For displacement of 3 mm the mean registration errors are 0.21±0.12 mm, 0.18±0.05 mm, and 0.25±0.08 mm in three axes, and for displacement of 5 mm their values are 0.11±0.06 mm, 0.21±0.08 mm, and 0.16±0.08 mm in three axes.

**Table 2:** Registration errors of DTS for cube phantom

	*Measured Shifts (mm)*					
*Displacement (mm)*	*DTS* $DTS(SR=20^{\circ })$	$DTS(SR=40^{\circ })$	$DTS(SR=60^{\circ })$
	*LAT‐LNG*	*LNG‐VRT*	*LAT‐LNG*	*LNG‐VRT*	*LAT‐LNG*	*LNG‐VRT*
0‐0‐0	(0.1, 0.0)	(0.2, 0.1)	(0.0, 0.1)	(0.1, 0.1)	(0.1, 0.1)	(0.0, 0.1)
1‐0‐0	(1.1, 0.5)	(0.4, 0.1)	(0.9, 0.2)	(0.2, 0.5)	(1.1, 0.1)	(0.1, 0.3)
3‐0‐0	(2.5, 0.2)	(0.1, 0.1)	(3.1, 0.2)	(0.1, 0.2)	(2.7, 0.3)	(0.2, 0.2)
5‐0‐0	(4.6, 0.3)	(0.1, 0.2)	(5.2, 0.2)	(0.1, 0.4)	(4.8, 0.2)	(0.0, 0.1)
0‐1‐0	(0.2, 1.2)	(1.1, 0.3)	(0.1, 0.9)	(0.8, 0.1)	(0.2, 1.1)	(1.3, 0.2)
0‐3‐0	(0.0, 2.7)	(3.2, 0.1)	(0.0, 2.9)	(3.1, 0.3)	(0.0, 2.9)	(3.0, 0.1)
0‐5‐0	(0.1, 4.8)	(4.8, 0.1)	(0.0, 4.9)	(4.7, 0.1)	(0.2, 5.2)	(5.1, 0.3)
0‐0‐1	(0.0, 0.1)	(0.2, 1.1)	(0.1, 0.0)	(0.1, 1.2)	(0.0, 0.1)	(0.0, 1.1)
0‐0‐3	(0.1, 0.0)	(0.1, 3.2)	(0.1, 0.0)	(0.0, 3.1)	(0.1, 0.0)	(0.1, 3.1)
0‐0‐5	(0.0, 0.0)	(0.0, 5.2)	(0.1, 0.0)	(0.1, 5.1)	(0.1, 0.1)	(0.1, 5.1)

SR=scan range;LAT=lateral;LNG=longitudinal;VRT=vertical.

**Table 3:** Registration errors of DTS for pelvis phantom

	*Measured Shifts (mm)*					
*Displacement (mm)*	*DTS* $DTS(SR=20^{\circ })$	$DTS(SR=40^{\circ })$	$DTS(SR=60^{\circ })$
	*LAT‐LNG*	*LNG‐VRT*	*LAT‐LNG*	*LNG‐VRT*	*LAT‐LNG*	*LNG‐VRT*
0‐0‐0	(0.1, 0.2)	(0.1, 0.1)	(0.2, 0.1)	(0.2, 0.1)	(0.1, 0.0)	(0.1, 0.2)
1‐0‐0	(0.8, 0.1)	(0.1, 0.2)	(0.9, 0.1)	(0.2, 0.1)	(0.9, 0.1)	(0.0, 0.1)
3‐0‐0	(3.1, 0.1)	(0.2, 0.3)	(3.3, 0.2)	(0.2, 0.3)	(2.9, 0.2)	(0.2, 0.3)
5‐0‐0	(5.2, 0.2)	(0.3, 0.2)	(4.7, 0.3)	(0.2, 0.2)	(5.1, 0.2)	(0.3, 0.1)
0‐1‐0	(0.0, 1.1)	(1.2, 0.2)	(0.1, 0.8)	(0.8, 0.2)	(0.2, 0.8)	(0.9, 0.1)
0‐3‐0	(0.3, 2.7)	(2.8, 0.3)	(0.1, 3.2)	(3.1, 0.2)	(0.1, 2.8)	(2.7, 0.3)
0‐5‐0	(0.0, 5.3)	(5.1, 0.3)	(0.1, 4.9)	(5.1, 0.4)	(0.0,5.2)	(5.2, 0.2)
0‐0‐1	(0.1, 0.2)	(0.3, 0.9)	(0.0, 0.2)	(0.1, 0.9)	(0.1, 0.3)	(0.3, 1.1)
0‐0‐3	(0.2, 0.2)	(0.1, 2.7)	(0.1, 0.2)	(0.3, 2.9)	(0.1, 0.2)	(0.1, 3.1)
0‐0‐5	(0.2, 0.2)	(0.3, 4.9)	(0.3, 0.1)	(0.3, 5.2)	(0.2, 0.3)	(0.3, 4.8)

SR=scan range;LAT=lateral;LNG=longitudinal;VRT=vertical.

### D. Reconstruction efficiency

The reconstruction efficiencies of DRR using three algorithms are compared for one reconstruction task in generating total 658 DRRs from a planning CT consisting of 150 slices. For CUDA‐based algorithm the reconstruction time is 200s (0.3 s for each DRR) and overhead time is 10 s. For OpenGL‐based algorithm, the reconstruction time is 40 s (0.06 s for each DRR) and overhead time is 13 s. Using CPU‐based algorithm, the total processing time is 1450 s (2.2 s for each DRR). The result shows that the performance of OpenGL‐based algorithm exceeds the performance of CUDA‐based algorithm, and the CPU‐based algorithm is the least efficient in accomplishing the whole reconstruction task. Compared with the processing time of CPU‐based algorithm, the performance of GPU‐based algorithms is boosted by a factor of 7 to 36 in term of the workload.

The performances of DTS reconstruction using three algorithms are compared in Table 4. Two parameters scan ranges (30°, 45°, and 60°) and the slice numbers (32, 64, 128, and 256), are investigated. The processing time increases linearly as the number of slices increased. This tendency is observed in all three algorithms. The processing time also increases as the scan range extended. For a scan range, the processing times of three algorithms are compared for all DTSs with different slice thicknesses. For scan range 30°, the mean processing times and their standard deviations are 0.53±0.16 s,0.41±0.13 s, and 0.25±0.02 s for CPU‐based, OpenGL‐based, and CUDA‐based algorithms, respectively. For scan range 45°, the mean processing times and their standard deviations are 0.74±0.16 s,0.41±0.31 s, and 0.26±0.01 s, and for scan range of 60° their values are 0.91±0.24 s,0.58±0.24 s, and 0.28±0.04 s. For two types of GPU algorithms, their reconstruction time and overhead time are compared separately. For OpenGL‐based algorithm, the mean reconstruction times and standard deviations are 0.25±0.01 s,0.28±0.01 s, and 0.30±0.01 s for scan range 30°, 45°, and 60°, respectively. And the mean overhead times and standard deviations are 0.16±0.13 s,0.22±0.17 s, and 0.28±0.26 s for scan range 30°, 45°, and 60°, respectively. While for CUDA‐based algorithm, the mean reconstruction times and standard deviations are 0.03±0.02 s,0.04±0.02 s, and 0.05±0.02 s for scan range 30°, 45°, and 60°, respectively. And the mean overhead times and standard deviations are 0.22±0.01 s,0.22±0.01 s, and 0.22±0.01 s for scan range 30°, 45°, and 60°, respectively. Compared with the processing time of CPU‐based algorithm, the performance of GPU‐based algorithms is improved by a factor of 10 to 22 in terms of the workload.

**Table 4:** Comparison of DTS reconstruction performance of three types of algorithms

	*Processing Time (s)*								
*Processing Time (CPU‐based algorithm)*			*Reconstruction‐Overhead Time (OpenGL‐based algorithm)*			*Reconstruction‐Overhead Time (CUDA‐based algorithm)*		
$N_{DTS}$	$N_{CBP}=55$ $\left(SR=30^{\circ }\right)$	$N_{CBP}=85$ $\left(SR=45^{\circ }\right)$	$N_{CBP}=115$ $\left(SR=60^{\circ }\right)$	$N_{CBP}=55$ $\left(SR=30^{\circ }\right)$	$N_{CBP}=85$ $\left(SR=45^{\circ }\right)$	$N_{CBP}=115$ $\left(SR=60^{\circ }\right)$	$N_{CBP}=55$ $\left(SR=30^{\circ }\right)$	$N_{CBP}=85$ $\left(SR=45^{\circ }\right)$	$N_{CBP}=115$ $\left(SR=60^{\circ }\right)$
$N_{\text{DTS}}=32$	24	31	40	8−11	9−15	9−21	2−7	2−7	3−8
$N_{\text{DTS}}=64$	38	49	60	16−12	18−14	19−19	2−14	3−14	4−14
$N_{\text{DTS}}=128$	55	82	100	32−10	35−15	40−15	3−29	4−29	6−28
$N_{\text{DTS}}=256$	102	150	180	69−10	76−18	81−20	4−56	7−56	8−57

$N_{\text{DTS}}=$ The number of DTS planes; SR=scan range; $N_{\text{CBP}}=$ the number of CB projections.

## IV. DISCUSSION

Based on the measurement of cube phantom on ODTS, the geometric error is within 0.5 mm in three axes. Compared with the measurement errors in lateral and vertical axes, its error in longitudinal axis is slightly more, but still within 0.5 mm. This may be caused by the uneven edge of cube phantom on ODTS in longitudinal axis. As shown in Fig. 6(a) and 6(d), both top and bottom edges of cube phantom are not flat. This was due to the reconstruction algorithm that the pixels near the vertical midline of DTS plane receives more weight than those of nearby. As a result, these pixels near the vertical midline show higher density than those at nearby in DTS plane. These pixels causes the edge of cube phantom on DTS planes looked uneven in longitudinal direction. As the number of CB projections increased, this effect is reduced as the difference between the densities of midline of DTS plane and the nearby become less. This is demonstrated in Figs. 6(i) and 6(l) which show that, as the number of CB projections increased, the edge of cube phantom in longitudinal axis is more flat than those in Figs. 6(a) and 6(d). Overall, the geometric accuracy of DTS is high enough to meet clinical requirement.

There is no apparent difference between DRRs generated by CPU‐based algorithm and GPU‐based algorithm based on the correlation analysis. The minor difference is observed from the comparison among profile plots of DRRs generated from three types of algorithms. For the horizontal profiles, the difference between DRRs generated by CPU‐based and GPU‐based algorithms is almost identical. For the vertical profiles, the difference is tiny, but visible. The profiles of DRRs generated by GPU‐based algorithms are coarser compared with those of DRRs generated by CPU‐based algorithm. This may be caused by the difference between interpolation mechanisms provided by CPU‐based and GPU‐based algorithms. In CPU‐based algorithm, the interpolation is performed on CPU and with trilinear interpolation. However in GPU algorithm, the interpolation is performed on GPU texture memory and with bilinear interpolation. Also, due to the limitation of GPU, floating operations might not be supported in certain functions of GPU‐based algorithm which cause loss of precision. Therefore, the DRRs generated by GPU‐based algorithms are coarser than those generated by CPU‐based algorithm. The same effect is observed in DTS generated by GPU‐based algorithms. As the relative means and standard deviations of subtraction images for DRRs and DTSs are 0.3%±0.2% and 0.4%±0.4%, these errors are trivial for registration.

The registration errors for both phantom cases are consistent and small. For the cube phantom and pelvis phantom, the registration errors are less than 0.5 mm in three axes. For different scan ranges, the registration errors are consistent and less than 0.3 mm in three axes. Also for different displacements of phantoms, the registration errors are less than 0.3 mm in three axes. This result is expected since registration of both cube phantom and pelvis phantom on DTS are mainly based on bony structures. For real patients, the influence of soft tissue and motion artifacts could be dominant and the registration accuracy could be decreased. The result of phantom studies indicates that the registration errors are less dependent on the scan ranges and phantom displacements. Therefore, with narrow scan range (such as 20°) the higher registration accuracy for certain cases with abundant bony structures (such as head‐and‐neck cases) could be possible. This is proved by many researchers in applying DTS registration for certain clinical treatment sites. In one report, DTS with scan angles of 20° in the coronal or sagittal views yields the same results for patient positioning as CBCT with positioning differences of <0.1 cm and 0.5°.^(9)^ For a DTS reconstructed with scan range of 20°, the total number of CB projections is 37×2=75 (two sets of CB projections in scan range of 20° are required and angle spacing is 0.53° per CB projection). Compared with the total number of CB projections (375) acquired by a full‐fan CBCT scan, DTS scan would save about 80% of total amount of CB projections. The reduction of imaging dose would be 1.6 cGy‐3.2 cGy per scan. For a typical IMRT treatment with 30 fractions, the accumulated dose reduction would be 48 cGy‐96 cGy. In other words, the imaging dose required by one CBCT scan is equal to the same imaging dose required by five DTS scans.

High‐performance reconstruction algorithms are critical for many online clinical applications. CUDA‐based techniques provided the best computation environment on general purpose computer. As demonstrated in the study, 256 slices of DTS in dimensions of 512×512 are reconstructed within 5‐8 s on middle‐level NVIDIA GPU, while producing the amount of slices on general‐purpose CPU would take 2‐3 min. However, it should be noted that the overhead of GPU algorithms could be considerably high. For OpenGL‐based DTS algorithm the reconstruction time is ∼0.3 s per slice and the overhead time is ∼0.25 s per slice. As shown in the Results section D, when projection data increased, the overhead time increases more than that of reconstruction time. This is because although in OpenGL algorithm the projection data is loaded into GPU texture memory at once, back‐projecting operation is performed in batch pattern which means the projections data are divided in multiple datasets and processed sequentially in order to accommodate the limited precision of GPU buffer.^26^, ^27^ Therefore, as the size of projection data increased, the processing time is increased proportionally. Similarly this effect is observed in OpenGL‐based DRR algorithm. In this case, the CT data are processed in batch pattern in order to accommodate the limited precision of GPU buffer. Since the CT slices are less than that of CB projections, the overhead time of DRR is less than that of DTS. For CUDA‐based DTS algorithm, the reconstruction time is far less than the overhead time. The overhead time is almost independent of the scan ranges and the size of projection data. As shown in Table 4, the reconstruction time is only 0.04 s per slice, but the overhead time is 0.22 s per slice. This is because the projection data are loaded into GPU memory at once and back‐projecting operations are performed at once instead of batch processing. But the preprocessing, including memory allocation and data transferring, in CUDA code is relatively slow and requires a lot of time. In summary, the GPU‐based algorithms are capable of improving reconstruction efficiency significantly, but the overhead time could be high if the reconstruction codes are not properly optimized.

## V. CONCLUSIONS

A software tool was developed to facilitate the clinical application of DTS in patient positioning in radiotherapy. The core reconstruction algorithms were implemented on GPU environment and their performance was evaluated by phantom studies. Based on the results, the registration accuracy is comparable to that of CBCT applications. It provides a feasible software tool for researcher and clinician in evaluating DTS using clinical cases.

## ACKNOWLEDGMENTS

The authors gratefully acknowledge the reviewers for their helpful comments. The author also wants to thank former colleagues Devon Godfrey, Ren Lei, Sua Yoo, and Fang‐Fang Yin at Department of Radiation Oncology, Duke University Medical Center, for their helpful discussion. In addition, the generous support from our colleagues Pan Ma, Kuo Men, and Yuan Tian at the Department of Radiation Oncology, Cancer Hospital Chinese Academy of Medical Sciences, Beijing, is highly appreciated.

## COPYRIGHT

This work is licensed under a Creative Commons Attribution 4.0 International License.

## APPENDICES

**Appendix A. The Pseudocodes for DRR Reconstruction Implemented on Three Computation Environments**

***Algorithm 1: CPU‐based DRR reconstruction***

(1) Allocate 3D array for CT volume and 2D array for DRR in CPU memory.
(2) Load CT volume into 3D array in CPU memory. **For** n=1:Num_of_DRRs
(3) Apply rotational transformation on 3D CT volume with the given angle. **For** k=1:Num_of_CT_coronal_planes
(4) Calculate intersecting points of ray with 2D slice of 3D CT volume.
(5) Interpolate the value of intersecting points along the ray.
(6) Sum the values of intersecting points on the ray
(7) Assign the value to pixel of 2D array of DRR.

**End**

(8) Save the 2D array as output file.

**End**

***Algorithm 2: OpenGL‐based DRR reconstruction***

(1) Allocate 3D array for CT volume and 2D array for DRR in CPU memory.
(2) Load CT volume into 3D array in CPU memory.
(3) Allocate 3D array for CT volume and 2D array for DRR in texture memory.
(4) Transfer 3D array into 3D texture array in GPU memory.

***For*** n=1:Num_of_DRRs

(5) Apply rotational transformation on 3D CT volume with the given angle.

**For** k=1:Num_of_CT_coronal_planes

(6) Apply projecting transformation on 2D CT coronal plane
(7) Accumulate it onto the frame buffer of GPU.
(8) If the content of frame buffer is full, transfer it to 2D array in CPU memory

**End**

(9) Save the 2D array as output file.

**End**

***Algorithm 3: CUDA‐based DRR reconstruction***

(1) Allocate 3D array for CT volume and 2D array for DRR in CPU memory.
(2) Load CT volume into 3D array in CPU memory.
(3) Allocate 3D array for CT volume and 2D array for DRR in texture memory.
(4) Transfer 3D array into 3D texture array in GPU memory.

***For*** n=1:Num_of_DRRs

**For** k=1:Num_of_DRR_Pixel

(5) Find intersecting points of ray at CT volume for pixels *k*.
(6) Get its CT value from 3D texture array.
(7) Apply proper scale factors to the CT value.
(8) Accumulate its values to pixel *k* in 2D array in GPU memory.

**End**

(9) Transfer resulting 2D array in GPU memory to 2D array in CPU memory.
(10) Save the 2D array as output file.

**End**

**Appendix B. The Pseudocodes for DTS Reconstruction Implemented on Three Computation Environments**

***Algorithm 1: CPU‐based DTS reconstruction***

(1) Allocate 3D array for DTS volume and 2D array for CB projections/DRRs in CPU memory.
(2) Load CB projection/DRR into 2D array in CPU memory.
(3) Perform logarithm conversion and FFT filtering on 2D CB projection/DRR. **For** n=1:Num_of_Projection

**For** m=1:Num_of_DTS_Plane

(4) Calculate intersecting point of ray in kV detector plane.
(5) Interpolate the value of intersecting point on 2D CB projection/DRR.
(6) Add the value to voxel of DTS on the ray.

**End**

**End**

(7) Save 3D array into multiple files of 2D DTS planes.

***Algorithm 2: OpenGL‐based DTS reconstruction***

(1) Allocate 3D array for DTS volume and 2D array for CB projections/DRRs in CPU memory.
(2) Load CB projection/DRR into 2D array in CPU memory.
(3) Perform logarithm conversion and FFT filtering on 2D CB projection/DRR.
(4) Allocate 3D array for DTS volume and 2D array for CB projections/DRRs in GPU memory.
(5) Transfer 2D CB projection/DRR into a stack of 2D texture in GPU memory.

**For** n=1:Num_of_Projection

**For** m=1:Num_of_DTS_Plane

(6) Apply translational and rotational transformation on 2D texture
(7) Apply projecting transformation 2D texture
(8) Accumulate it on the frame buffer of GPU.
(9) If the content of frame buffer is full, transfer it to 2D array allocated on CPU memory

**End**

**End**

(10) Save 3D array into multiple files of 2D DTS planes.

***Algorithm 3: CUDA‐based DTS reconstruction***

(1) Allocate 3D array for DTS volume and 2D array for CB projections/DRRs in CPU memory.
(2) Load CB projection/DRR into 2D array in CPU memory.
(3) Perform logarithm conversion and FFT filtering on 2D CB projection/DRR.
(4) Allocate 3D array for DTS volume and 2D array for CB projections/DRRs in GPU memory.
**For** n=1:Num_of_Projection
(5) Transfer 2D CB projections/DRRs into 2D texture memory in GPU.
**For** k=1: Num_of_DTS_Voxel
(6) Find the index of pixel at *n*‐th 2D CB projections/DRRs for *k*‐th voxel of DTS volume.
(7) Get the value of pixel from 2D array in texture memory.
(8) Apply proper scale factor and accumulate this value on *k*‐th voxel in GPU memory.

**End**

**End**

(9) Transfer resulting 3D DTS array back to 3D array in CPU memory.
(10) Save 3D array into multiple files of 2D DTS planes.
